# The effect of repeated hot water immersion on microvascular function, glycaemic control and inflammation in White European and South Asian males

**DOI:** 10.1113/EP093305

**Published:** 2026-02-17

**Authors:** David Bellini, Alex Lloyd, Christof A. Leicht, Stephen J. Bailey, Lewis J. James, Matthew J. Maley

**Affiliations:** ^1^ Environmental Ergonomics Research Centre Loughborough University Loughborough UK; ^2^ School of Sport, Exercise, and Health Sciences Loughborough University Loughborough UK; ^3^ The Peter Harrison Centre for Disability Sport Loughborough University Loughborough UK; ^4^ NIHR Leicester Biomedical Research Centre University Hospitals of Leicester NHS Trust and the University of Leicester Leicester UK

**Keywords:** glycaemic control, heat therapy, microvascular function

## Abstract

Individuals of South Asian (SA) descent display a higher risk for cardiovascular disease and type 2 diabetes mellitus than their White European (WE) counterparts. Heat therapy, such as hot water immersion (HWI), can improve microvascular function and glycaemic control, although effects across racial groups are unknown. This study compared how repeated HWI influenced microvascular function, glycaemic control and markers of inflammation in males of WE and SA descent. Ten WE and SA males completed ten 60 min HWI sessions over 14 days. Before and after HWI, forearm and great toe cutaneous vascular conductance (CVC) responses to postocclusive reactive hyperaemia and local heating (LH) were measured, resting blood samples were collected, and an oral glucose tolerance test (OGTT) was conducted. Baseline great toe CVC did not differ between racial groups (*P* = 0.670), but forearm CVC was lower in SA (*P* = 0.010). For postocclusive reactive hyperaemia, forearm and great toe peak CVC and area under the curve were unchanged by HWI (*P* ≥ 0.300), whereas CVC during 42°C LH increased at both sites after HWI (*P* ≤ 0.037), as did great toe CVC during 44°C LH (*P* = 0.021). Glucose and insulin concentrations were elevated in SA during the OGTT (*P* ≤ 0.035); glucose concentration and peak insulin reduced after HWI in SA (*P* ≤ 0.024), but not in WE. The interleukin‐6:interleukin‐10 ratio was unchanged by HWI (*P* = 0.159), but elevated in SA (*P* = 0.006). Repeated HWI increased microvascular responses to LH to a similar extent between racial groups and reduced early‐phase OGTT glucose concentrations in SA, although insulin sensitivity was unchanged. These findings support HWI as a health‐promoting intervention.

## Abstract (highlights)

- **What is the central question of this study?**
  Are there racial differences in the effects of repeated hot water immersion on microvascular function, glycaemic control and inflammatory markers between White European and South Asian males?
- **What is the main finding and its importance?**
  Hot water immersion increased microvascular responses to cutaneous local heating to a similar extent between racial groups and reduced glucose concentrations during an oral glucose tolerance test in South Asians, although insulin sensitivity was unchanged. These findings support hot water immersion as a health‐promoting intervention, particularly for South Asian males, who typically present with elevated microvascular and metabolic dysfunction.

## 1 INTRODUCTION

Atherosclerotic cardiovascular disease (CVD) is the leading global cause of death (Martin et al., 2024). Individuals of South Asian (SA) descent display a greater CVD morbidity and mortality in comparison to White European (WE) counterparts (Office for National Statistics, 2023), with prior studies estimating a 1.7‐ to 4‐fold higher risk of CVD (Patel et al., 2021, 2025). In the UK, SA individuals are at higher risk of coronary heart disease (George et al., 2017; Kuppuswamy & Gupta, 2005), with an onset earlier in life (Ho et al., 2022). Likewise, SA individuals are three to five times more likely to present with type 2 diabetes mellitus (T2DM) than WE individuals (Goff, 2019), in addition to exhibiting elevated markers of inflammation and oxidative stress (Brady et al., 2012; Chambers et al., 2001). Together, inflammation and oxidative stress promote endothelial dysfunction by attenuating the production and bioavailability of nitric oxide (NO) (Libby et al., 2002; Najjar et al., 2005) and contribute to insulin resistance by impairing insulin signalling, glucose uptake and β‐cell function (An et al., 2023). Although both SA males and females display elevated cardiometabolic risk profiles relative to WE counterparts, within each racial group the prevalence of both CVD (Patel et al., 2021) and T2DM (Kanaya et al., 2014) is greater in males than females. This increased disease burden within males has been attributed to greater visceral fat accumulation and central adiposity (Ho et al., 2022; Meeks et al., 2016), in addition to heightened insulin resistance (Goff, 2019) and more adverse lipid profiles (Patel et al., 2021).

Vascular endothelial function is a prognostic factor for CVD morbidity and mortality, with dysfunction often considered the initial stage of development of atherosclerosis (Lind et al., 2011; Yeboah et al., 2009). Impaired macro‐ and microvascular responses to stimuli such as occlusion, infusion of vasodilators or local heating (LH) reflect endothelial dysfunction (Najjar et al., 2005) and might provide insight into the racial disparity in CVD. Although SA have exhibited reduced flow‐mediated dilatation (Chambers et al., 1999; Murphy et al., 2007; Roberts et al., 2023; Shantsila et al., 2011) and impaired microvascular responses to acetylcholine (Shantsila et al., 2011) in comparison to WE, other studies have reported comparable responses between these racial groups (Bellini et al., 2025). Although relatively limited experimental research has compared vascular function directly between WE and SA populations, differences in glycaemic control and insulin resistance are well reported. For example, SA individuals have elevated glycated haemoglobin (Hb_A1c_) (Mostafa et al., 2012), and insulin resistance is a commonly reported characteristic of SA individuals with and without T2DM (Mohan et al., 1986).

Heat therapy (HT) involves the passive elevation of body core temperature and has been used by various cultures throughout history (Jackson, 1990). There is evidence that HT is associated with reduced all‐cause and cardiovascular‐related mortality (Laukkanen et al., 2018, 2015), with the potential to extend health span (Laukkanen & Kunutsor, 2024). Indeed, HT has received attention as a health‐promoting intervention, given the positive effects on cardiovascular responses (Brunt & Minson, 2021; Cheng & MacDonald, 2019; Pizzey et al., 2021) and the emerging experimental evidence suggesting benefits for outcomes related to T2DM (Laukkanen & Kunutsor, 2024; Sebők et al., 2021). Specifically, HT has improved macrovascular and blood pressure (BP) responses (Bailey et al., 2016; Brunt, Howard, Francisco, et al., 2016; Carter et al., 2014a; Ely, Francisco, Halliwill, et al., 2019; Kaluhiokalani et al., 2024; Roxburgh et al., 2023; Ruiz‐Pick et al., 2025), in addition to microvascular function (Brunt, Eymann, Francisco, et al., 2016; Carter et al., 2014b; Green et al., 2010). Moreover, markers of glucose metabolism, such as fasting glucose and insulin (Hoekstra et al., 2018; James et al., 2023; Koçak et al., 2020; Pallubinsky et al., 2020) and Hb_A1c_ (Hooper, 1999), have been reduced by HT, and improvements in glycaemic control (Ely, Clayton, McCurdy, et al., 2019; Fuchs et al., 2025) and insulin sensitivity (James et al., 2023) are documented. Importantly, previous studies have suggested that individuals exhibiting impaired health profiles might experience a heightened benefit from HT (Hoekstra et al., 2018; James et al., 2023). However, research into HT has overwhelmingly included WE individuals, with limited comparison or inclusion of non‐White racial groups. Given the potential of HT to improve outcomes related to CVD and T2DM, alongside the heightened risk profiles of these chronic diseases within the SA population, including SA individuals within HT research is both relevant and warranted.

To our knowledge, no study to date has compared the chronic responses to HT between WE and SA racial groups. Therefore, the aim of this study was to investigate the effect of repeated hot water immersion (HWI) on microvascular function, glycaemic control and inflammatory markers between males of WE and SA descent. It was hypothesized that SA would have reduced microvascular function and glycaemic control, in addition to elevated inflammation, compared with WE, and although both racial groups would receive benefits from HWI, improvements would occur to a greater extent in males of SA descent. For any race‐specific responses from HT, whether distinct effects were related to baseline population differences was explored subsequently.

## 2 MATERIALS AND METHODS

### 2.1 Ethical approval

Experimental procedures were approved by the ethics committee of the institution (#17344) and conformed to the *Declaration of Helsinki* (1964) in all aspects apart from registration in a database, with all participants providing written and informed consent.

### 2.2 Participants

A total of 20 male participants were recruited, consisting of 10 individuals of WE descent and 10 individuals of SA descent (Table 1). Racial groups were defined based on the birthplace of participants’ grandparents, a method used previously within physiology research (Birt et al., 2010). Specifically, all four grandparents were either White and born in Europe (WE) or born in South Asia (SA), defined as Afghanistan, Bangladesh, Bhutan, India, Maldives, Nepal, Pakistan and Sri Lanka. Only males were included in this research to minimize potential biological variability associated with sex differences and hormonal environments (Charkoudian et al., 1999; Su et al., 2025; Turner et al., 2023), particularly given that research characterizing these influences has primarily involved females of WE descent. Participants self‐reported as recreationally active (≥30 min of moderate‐intensity exercise at least three times per week for the last 3 months), non‐smoking, not currently taking prescription medication and free from any cardiovascular, metabolic or skin conditions.

**TABLE 1:** Participant characteristics for groups of White European and South Asian descent.

Characteristic	WE (*n* = 10)	SA (*n* = 10)
Age, years	23 ± 5	24 ± 2
Height, cm	179.9 ± 2.3^#^	174.3 ± 4.7
Body mass, kg	83.0 ± 8.3	75.3 ± 10.7
Body mass index, kg/m^2^	25.6 ± 2.5	24.8 ± 3.2
Body fat, %	17.6 ± 4.4	20.0 ± 3.8
Body surface area, m^2^	2.03 ± 0.10^#^	1.89 ± 0.13
Estimated maximal O_2_ uptake, mL/min/kg	48 ± 8	46 ± 10

*Notes*: Data are the mean ± SD. Abbreviations: SA, South Asian; WE, White European.

^#^ Significant main effect of race (*P* < 0.05).

### 2.3 Experimental design

**FIGURE 1:**
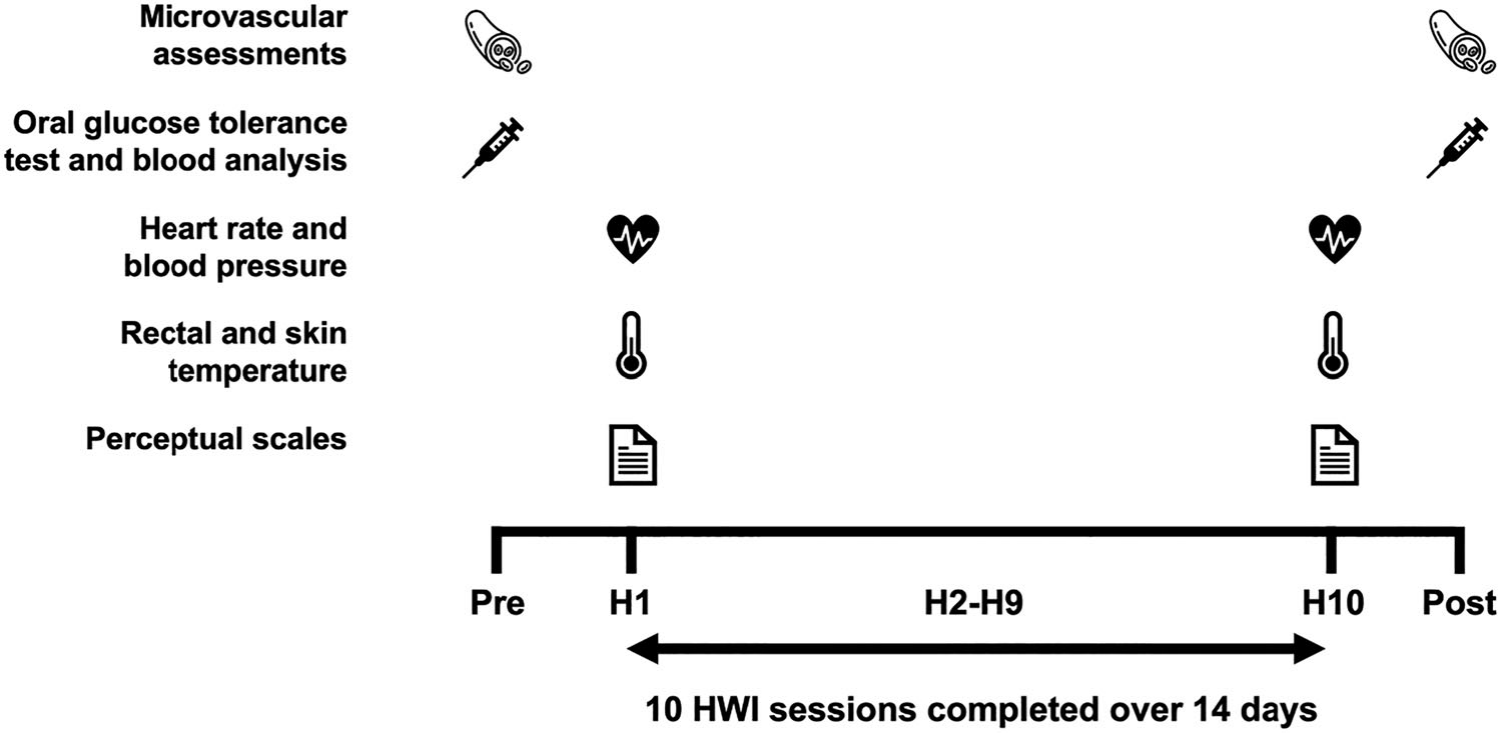
Schematic overview of experimental procedures. Participants completed visits before (Pre) and after (Post) the intervention, separated by 10 hot water immersion (HWI) sessions (H1–H10).

A pre–post study design was used, whereby an initial screening visit involved participants completing a health‐screen questionnaire and providing written informed consent, in addition to estimating fitness levels through the 6 min Åstrand–Rhyming submaximal cycling test (Lode Excalibur, Groningen, the Netherlands) using heart rate (HR), as previously described (Bellini et al., 2025). During the first experimental visit (Pre), microvascular function and glycaemic control, the primary outcome measures of this study, were assessed (Figure 1). Over the course of 14 days, participants then completed 10 HWI sessions, with microvascular function and glycaemic control re‐assessed within 24–48 h after the final HWI session (Post). A maximum of one HWI session occurred on any single day, with the start time self‐selected by participants for logistical purposes. The start time for the Pre and Post experimental visits commenced between 08.00 and 09.00 h, after an overnight fast of ≥12 h. A food diary was administered, where participants recorded the amounts of food and liquids consumed in the 24 h before Pre and were asked to replicate this for the Post experimental session. Participants refrained from alcohol, caffeine, vigorous activity, foods high in nitrate and antibacterial mouthwash for 24 h prior to the Pre and Post experimental visits and were asked to maintain habitual physical activity and diet throughout the HWI intervention.

### 2.4 Pre/post experimental visits

For Pre and Post experimental visits, euhydration was confirmed as urine specific gravity ≤1.020 (Master Refractometer, ATAGO, Japan) (Armstrong et al., 1994). Thereafter, height (Invicta, Bishop, UK) and body mass (KCC150, Mettler Toledo, USA) were measured, followed by body fat percentage through four‐point bioelectrical impedance (MC‐780 MA, Tanita, Japan). Body surface area was calculated using the Du Bois and Du Bois equation (Du Bois & Du Bois, 1989). Participants then commenced 10 min of seated rest in an environmentally controlled laboratory. During this time, four wireless thermochrons (DS1922L‐F50, Maxim Integrated, USA) were secured to the chest, triceps, thigh and shin using a single piece of adhesive tape (Hypafix, Leukoplast, UK) for calculation of mean skin temperature (*T*_skin_) (Ramanathan, 1964). Once supine, a BP cuff was then fitted on the right arm, and an occlusion cuff was positioned as proximal as possible on the left arm (SC10D, Hokanson, USA) in addition to a cuff around the base of the left Great toe (UDC2.5, Hokanson, USA). Following an initial BP and HR measurement (Tango M2, SunTech Medical, USA), flux measurement, an index of skin blood flow, of the left forearm and left great toe pad commenced through a single‐fibre laser Doppler probe (VP12, Moor Instruments, UK) within an integrated heater (VHP2, Moors Instruments, UK) connected to a laser Doppler perfusion monitor (moor VMS‐LDF2, Moor Instruments, UK). A combination of site measuring and ink markings was used to ensure that probe locations were as similar as possible across visits, avoiding placement over visible veins. Laser Doppler flux was recorded at 40 Hz and was averaged per second, with movement artefacts removed through linear interpolation and noise filters using computer software (MoorVMS‐PC v.4.2, Moor Instruments, UK). Throughout skin blood flow assessments, BP was measured every 5 min from the right brachial artery to calculate mean arterial pressure (MAP) as:

$$\mathrm{MAP}=\mathrm{DBP}+\frac{1}{3}\times \left(\mathrm{SBP}-\mathrm{DBP}\right)$$

Resting systolic (SBP) and diastolic (DBP) blood pressure, MAP and HR were determined as the average of the first two readings during postocclusive reactive hyperaemia (PORH). Flux data were converted to cutaneous vascular conductance (CVC) by dividing flux by MAP.

### 2.5 Postocclusive reactive hyperaemia

Cutaneous PORH was assessed in thermoneutral conditions [24.3°C ± 0.7°C; 42.7% ± 6.4% relative humidity (RH)] (Testo 435, Testo SE, Germany) with a protocol used previously (Bellini et al., 2025; Eglin et al., 2023). Briefly, local skin temperature was held constant at 33°C using the integrated heater throughout PORH. Following 10 min of baseline measurements, occlusion cuffs were rapidly inflated to 220 mmHg for 5 min through a pressure cuff controller (Moor‐VMS‐Pres, Moor Instruments, UK). Thereafter, occlusion cuffs were rapidly deflated, and the cutaneous PORH response was measured for a further 10 min. Baseline CVC was defined as the mean CVC of the last 5 min prior to occlusion, and the peak response during PORH was measured as the maximum CVC following occlusion release. The area of hyperaemia was determined as the CVC area under the curve (AUC) between the release of occlusion and returning to baseline, whereas the PORH index was calculated by dividing the CVC AUC of the first 1 min following occlusion release by the AUC from the final 1 min prior to inflation. The PORH index is not as commonly used as the AUC and peak when characterizing the PORH response, resulting in limited research on the between‐day repeatability. Nonetheless, it has been reported as an independent determinant of CVD in T2DM patients (Yamamoto‐Suganuma & Aso, 2009), in addition to being lower in individuals with a history of coronary artery disease in comparison to healthy control subjects, while displaying similar between‐day repeatability to the more established PORH measures (Çekiç et al., 2017).

### 2.6 Local heating

Immediately after completion of PORH, the cutaneous LH protocol commenced, as previously described (Bellini et al., 2025; Eglin et al., 2023) in similar ambient conditions (24.3°C ± 0.9°C; 41.4% ± 6.1% RH). During the first 11 min, local skin temperature was clamped at 33°C at the same locations as PORH. Thereafter, probe temperature increased by 0.1°C/s to 42°C and was maintained for 21 min 30 s, then increased at the same rate until 44°C for 10 min 20 s. To calculate 42°C and 44°C LH plateaus, CVC was averaged over the final 5 min of heating for each temperature. Although 42°C is an LH temperature commonly used when quantifying CVC plateaus (Minson et al., 2002), the CVC values for some participants might still have been increasing during the plateau stage, although similar heating durations (20–30 min) have been used to achieve plateaus (Eglin et al., 2023; Roustit et al., 2010). Although previous research has reported moderate‐to‐low repeatability of CVC measures during LH using single‐fibre measurement probes, attributed to anatomical variability within the microcirculation of the skin (McGarr et al., 2017), there is higher repeatability in glabrous (e.g., toe pad) than non‐glabrous (e.g., forearm) skin sites (Roustit et al., 2010), in addition to higher repeatability when converting raw flux values to CVC (Cracowski et al., 2006).

### 2.7 Venous blood sampling and oral glucose tolerance test

After completion of LH, an oral glucose tolerance test (OGTT) was conducted, with participants remaining seated for the entire duration. Initially, a venous blood sample from the antecubital vein was collected into a 6 mL lithium heparin tube (Vaccutainer, BD, USA), followed by collection of a 500 µL capillary blood sample. Thereafter, participants consumed 75 g of glucose (dextrose monohydrate, Myprotein, UK) in 300 mL of water within 5 min. Throughout the 120 min OGTT, capillary blood samples were collected at 15, 30, 45, 60, 90 and 120 min following ingestion. Although venous plasma glucose is recommended for clinical diagnosis of T2DM, capillary sampling is commonly used in point‐of‐care testing, and fasting and postprandial capillary glucose have shown good correlation with venous (Tan et al., 2021) and arterial (Förster et al., 1972) values.

### 2.8 Hot water immersion

During the first HWI session (H1), participants initially completed 10 min of seated rest, during which BP and HR were measured in duplicate. Thereafter, thermal comfort (TC) and thermal sensation (TS) were reported using a 30 cm visual analog scale, with 30 corresponding to ‘very comfortable’ and ‘very hot’ and 0 reflecting ‘very uncomfortable’ and ‘very cold’ for TC and TS, respectively. A thermistor (YSI 400, DeRoyal, USA) was self‐inserted ∼12 cm beyond the anal sphincter and connected to a datalogger (Squirrel 2010, Grant Instruments, UK) to record rectal temperature (*T*_core_). Using the same method as for the Pre and Post trials, *T*_skin_ was recorded throughout HWI, as was *T*_core_.

Thereafter, participants entered the water bath (Lay‐Z‐Spa St. Lucia, Bestway, UK) and were immersed to the clavicle while sitting semi‐supine in 39.0°C ± 0.2°C water, which was continuously circulated to maintain a uniform temperature. Following 30 min of immersion, participants sat upright, were towel‐dried above the waist and then immersed to the umbilicus for a further 30 min with their arms at heart level. Every 15 min throughout immersion, TC and TS were measured, whereas BP was measured from the right brachial artery at 30, 45 and 60 min of immersion. To compare acute responses across the intervention, this was repeated for the final HWI session (H10).

### 2.9 Blood analysis

Initially, 20 µL of capillary whole blood was analysed directly for [glucose] (Biosen C‐Line, EKF Diagnostics, UK) at all specified collection time points. The remaining venous and capillary blood was immediately centrifuged at 3500*g* (Medifuge, Thermo Fisher Scientific, USA) for 10 min, with the resulting plasma stored at −80°C until batch analysis. Capillary plasma [insulin] was measured using a commercially available ELISA kit (Mercodia AB, Sweden), as were venous fasting interleukin‐6 (IL‐6; Biotechne, USA) and interleukin‐10 (IL‐10; ThermoScientific, UK), using a microplate reader (Varioskan Flash, ThermoScientific, UK). The intra‐assay coefficient of variation for insulin (3.9%), IL‐6 (4.6%) and IL‐10 (1.8%) was determined through duplicate analysis, with all samples from the same participant and an equal number of WE and SA participants analysed on the same microplate.

Total incremental areas under the curve (iAUC) for capillary whole‐blood glucose and plasma insulin were calculated using the trapezoidal rule, with time point 0 (fasted) as the baseline, in addition to the early‐phase iAUC (0–30 min), late‐phase iAUC (30–120 min), mean and peak throughout the OGTT, and the Matsuda index served as a proxy for whole‐body insulin sensitivity (Matsuda & DeFronzo, 1999). Venous plasma [nitrite] was determined through ozone chemiluminescence using the same method as previously described (Bellini et al., 2025).

### 2.10 Statistical analysis

An a priori power calculation was conducted to determine the number of participants required to observe a significant interaction effect between race (two levels) and HWI (two levels) in G*Power. Effect sizes were incorporated from HT research investigating microvascular function (Brunt, Eymann, Francisco, et al., 2016; *d* = 0.76) and glycaemic control (Hoekstra et al., 2018; *d* = 1.06) as outcome measures. Using the smaller effect size, a power of 0.8, correlation between repeated measures of 0.5, non‐sphericity correction of 1.0 and an α of 0.05 yielded a total sample size of 20 participants, resulting in 10 per racial group for a repeated‐measures design.

All statistical analysis was completed using SPSS (v.28, IBM, USA). All data were assessed for normality using the Shapiro–Wilk test, and and a logarithmic transformation was performed when non‐normality was detected. Logarithmic transformation was performed on IL‐6, IL‐10 and IL‐6:IL‐10 ratio data. A one‐way ANOVA was used to compare anthropometric and estimated fitness levels between races. The effect of HWI on microvascular responses, glycaemic control outcomes, plasma nitrite, IL‐6 and IL‐10 between racial groups was assessed using a two‐way mixed‐model ANOVA, with race (WE, SA) as the between‐subject factor and HWI (Pre, Post) as the within‐subject factor, including the race × HWI interaction. To investigate [glucose] and [insulin] responses throughout the OGTT, a three‐way mixed‐model ANOVA, including race (WE, SA), HWI (Pre, Post) and time (0, 15, 30, 45, 60, 90 and 120 min), was used. To determine differences in thermoregulatory, cardiovascular and perceptual responses throughout immersion, a three‐way mixed‐model ANOVA, with race (WE, SA), HWI session (H1, H10) and time (0, 30, 60 min), was conducted. When significant effects were observed, differences within racial groups were examined using unadjusted *post hoc* pairwise comparisons. Effect sizes are reported as partial eta squared (*η*_p_^2^), with magnitudes of ≥0.01, ≥0.059 and ≥0.138 interpreted as small, medium and large, respectively (Cohen, 1988). To assess whether race‐specific responses were explained by baseline differences, a one‐way ANCOVA was conducted, in which Pre–Post change in outcome measure from HWI was included as the dependent variable, with the ‘Pre’ value as a covariate and race as a fixed factor. For all analyses, the statistical significance threshold was set at *P* < 0.05. All data are presented as the mean ± SD.

## 3 RESULTS

### 3.1 Participants

Participant characteristics are presented in Table 1. The only differences between racial groups were greater height (*P* = 0.005) and body surface area (*P* = 0.024) in WE.

### 3.2 Postocclusive reactive hyperaemia

Baseline forearm and great toe CVC were unchanged by HWI (Table 2; all *P* ≥ 0.524), although SA had lower baseline forearm CVC than WE (*P* = 0.010; *η*_p_^2^ = 0.327) but similar baseline great toe CVC (*P* = 0.672). Forearm and great toe peak CVC (all *P* ≥ 0.300), CVC AUC (all *P* ≥ 0.549) and PORH index (all *P* ≥ 0.677) were unchanged across the HWI intervention. Forearm PORH index was higher in SA (*P* = 0.027; *η*_p_^2^ = 0.243) with no race × HWI interaction effects for any PORH outcome (all *P* ≥ 0.079).

**TABLE 2:** Forearm and creat toe cutaneous vascular conductance responses during postocclusive reactive hyperaemia before (Pre) and after (Post) hot water immersion for groups of White European and South Asian descent.

Parameter	Forearm				Great toe			
	WE		SA		WE		SA	
	Pre	Post	Pre	Post	Pre	Post	Pre	Post
Baseline CVC, flux/mmHg	0.15 ± 0.05	0.19 ± 0.09	0.12 ± 0.04^#^	0.10 ± 0.02^#^	0.95 ± 0.51	0.83 ± 0.45	0.99 ± 0.59	0.97 ± 0.52
Peak CVC, flux/mmHg	0.74 ± 0.15	0.97 ± 0.21	0.77 ± 0.33	0.71 ± 0.19	3.17 ± 0.82	3.33 ± 0.76	3.27 ± 0.68	3.34 ± 0.93
Area under the curve, CVC × s	50.95 ± 17.98	47.11 ± 17.28	42.19 ± 18.21	40.09 ± 25.36	291.11 ± 107.00	259.07 ± 138.91	301.57 ± 206.88	281.26 ± 116.85
PORH index, CVC	3.64 ± 1.37	3.54 ± 1.14	4.61 ± 1.48^#^	4.95 ± 1.19^#^	2.98 ± 1.29	4.35 ± 2.69	4.07 ± 2.71	3.21 ± 2.09

*Notes*: Data are mean ± SD. PORH forearm CVC SA (*n* = 9). Abbreviations: AUC, area under the curve; CVC, cutaneous vascular conductance; PORH, postocclusive reactive hyperaemia; SA, South Asian; WE, White European.

^#^ Significant main effect of race (*P* < 0.05).

### 3.3 Cutaneous local heating

**FIGURE 2:**
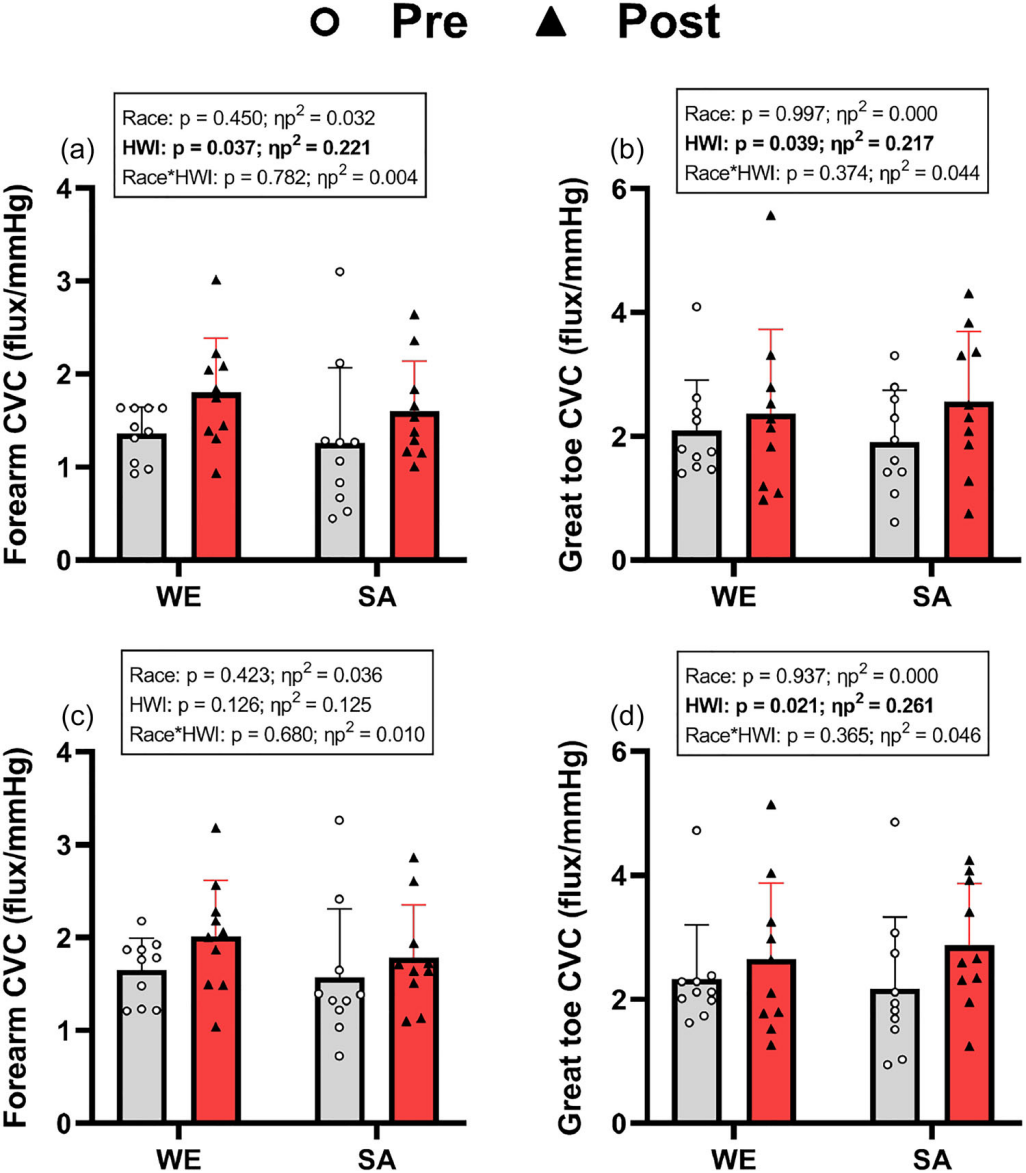
Forearm and great toe cutaneous vascular conductance (CVC) during 42°C (a, b) and 44°C (c, d) local heating before (Pre; grey bars) and after (Post; red bars) hot water immersion (HWI) for groups of White European (WE) and South Asian (SA) descent. Data are mean ± SD.

Forearm and great toe CVC during 42°C LH increased Post compared with Pre HWI (Figure 2a, b). This improvement also extended to the great toe during 44°C LH, which was elevated Post HWI (Figure 2d), and although non‐significant, there was a medium effect size for the difference between Pre and Post 44°C LH forearm CVC (Figure 2c). Forearm and great toe CVC were similar between races (all *P* ≥ 0.423) with no race × HWI interaction effects (all *P* ≥ 0.365).

### 3.4 Glucose and insulin

**FIGURE 3:**
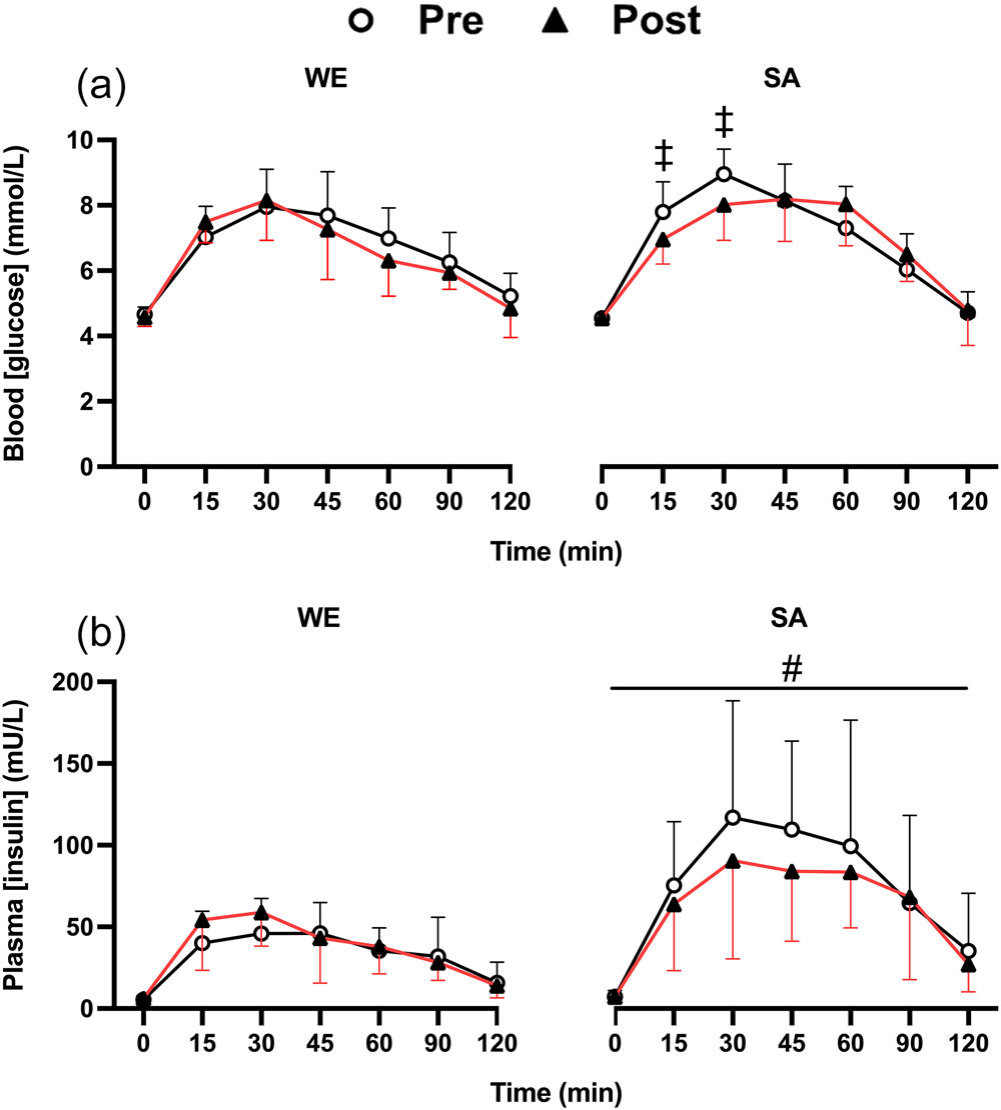
Glucose (a) and Insulin (b) during the oral glucose tolerance test before (Pre; black lines) and after (Post; red lines) hot water immersion for groups of White Europeans (WE) and South Asian (SA) descent. ^#^Significant main effect of race (*P* < 0.05). ^‡^Significant difference between Pre and Post within racial group (*P* < 0.05). Data are the mean ± SD.

For capillary blood [glucose], there were no significant main effects of HWI, race or a race × HWI interaction (all *P* ≥ 0.194; Figure 3a). However, a significant HWI × race × time interaction (*P* < 0.001; *η*_p_^2^ = 0.262) indicated that HWI affected the glucose response differently between racial groups throughout the OGTT. Specifically, although SA had elevated [glucose] compared with WE at 30 min Pre HWI (*P* = 0.035) and at 60 min Post HWI (*P* = 0.005), Post HWI values were reduced at 15 min (*P* = 0.011) and 30 min (*P* = 0.019) of the OGTT compared with Pre in SA, whereas there were no significant differences in WE. Consequently, glucose iAUC 0–30 min was reduced in SA (*P* = 0.002) but not WE (*P* = 0.074; Table 3).

**TABLE 3:** Glucose and insulin responses throughout the oral glucose tolerance test before (Pre) and after (Post) hot water immersion for groups of White European and South Asian descent.

Parameter	Glucose (mmol/L)				Insulin (mU/L)			
	WE		SA		WE		SA	
	Pre	Post	Pre	Post	Pre	Post	Pre	Post
Mean	6.54 ± 0.60	6.37 ± 0.60	6.78 ± 0.56	6.72 ± 0.45	32 ± 11	35 ± 10	73 ± 37^#^	61 ± 26^#^
Peak	8.26 ± 0.93	8.32 ± 1.02	9.00 ± 0.78	8.78 ± 0.87	55 ± 17	71 ± 28	147 ± 70^#^	114 ± 46^#^, ^‡^
iAUC 0–120 min	239 ± 76	221 ± 64	278 ± 75	284 ± 54	3358 ± 1360	3557 ± 1389	8418 ± 4757^#^	7087 ± 3485^#^
iAUC 0–30 min	60 ± 19	71 ± 14	82 ± 15	62 ± 13^‡^	824 ± 357	1115 ± 543	1845 ± 999^#^	1473 ± 885^#^
iAUC 30–120 min	178 ± 62	151 ± 55	196 ± 66	222 ± 57	2534 ± 1177	2442 ± 1114	6573 ± 4082^#^	5613 ± 2903^#^

*Notes*: Data are the mean ± SD. Abbreviations: iAUC, incremental area under the curve; SA, South Asian; WE, White European.

^#^ Significant main effect of race (*P* < 0.05).

^‡^ Significant difference between Pre and Post within racial group (*P* < 0.05).

For capillary plasma [insulin], there was a significant main effect of race, whereby SA had elevated [insulin] during the OGTT (*P* = 0.005; *η*_p_^2^ = 0.368), although there was no significant main effect of HWI, HWI × race or HWI × race × time interaction (all *P* ≥ 0.115; Figure 3b). As a result, there was greater mean, peak, iAUC, iAUC 0–30 min and iAUC 30–120 min plasma [insulin] in SA compared with WE (all *P* ≤ 0.044; *η*_p_^2^ ≥ 0.206; Table 3). However, peak plasma [insulin] was reduced Post HWI in SA (*P* = 0.024) but not in WE (*P* = 0.249), and despite a significant race × HWI interaction effect (*P* = 0.034; *η*_p_^2^ = 0.227), there were no differences in insulin iAUC 0–30 min between Pre and Post HWI for either racial group (all *P* ≥ 0.085).

The ratio of capillary [glucose]:[insulin] during the OGTT was greater, and whole‐body insulin sensitivity (as determined by the Matsuda index) was lower in SA (all *P* ≤ 0.018; *η*_p_^2^ ≥ 0.274), these were unchanged by HWI (all *P* ≥ 0.435), and there were no race × HWI interactions (all *P* ≥ 0.235). Lastly, for variables where significant race × HWI interaction effects occurred, baseline values did not influence the response to HWI differently between racial groups for either glucose iAUC 0–30 min (*P* = 0.915; *η*_p_^2^ = 0.001) or peak insulin (*P* = 0.760; *η*_p_^2^ = 0.006).

### 3.5 Inflammatory and nitrite responses

**FIGURE 4:**
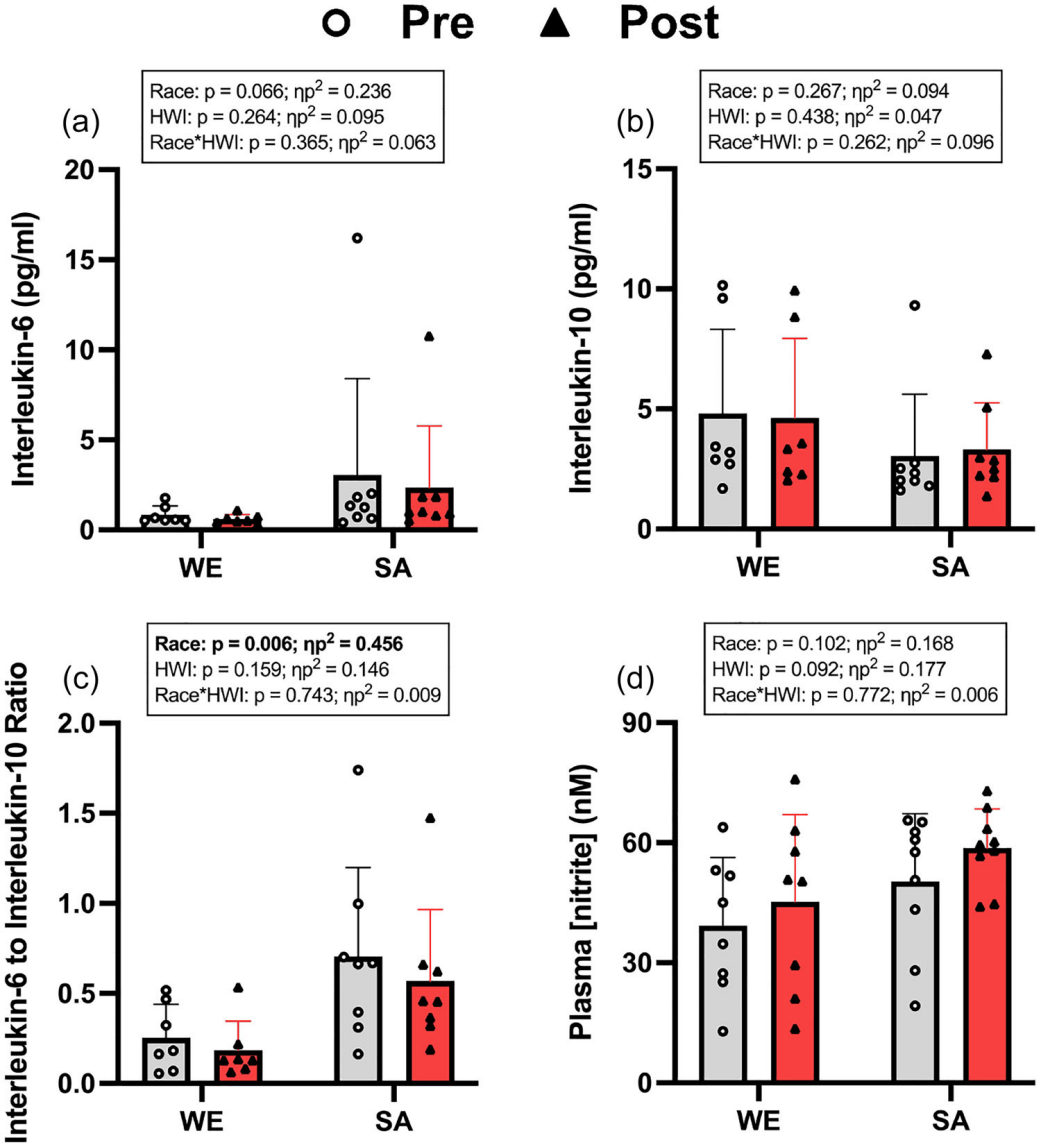
The plasma interleukin‐6 (a), interleukin‐10 (b), interleukin‐6 to interleukin‐10 ratio (c) and nitrite (d) concentrations before (Pre; grey bars) and after (Post; red bars) hot water immersion sessions for groups of White European (WE; IL‐6 and IL‐10 *n* = 7, nitrite *n* = 8) and South Asian (SA; IL‐6 and IL‐10 *n* = 8, nitrite *n* = 9) descent. Data are the mean ± SD.

Although plasma IL‐6 and IL‐10 were not different between racial groups (all *P* ≥ 0.066), the IL‐6:IL‐10 ratio was elevated in SA (*P* = 0.006) (Figure 4a–c). Inflammatory markers were unchanged from HWI (all *P* ≥ 0.159), but there was a large effect size for the reduction of the IL‐6:IL‐10 ratio (*η*_p_^2^ = 0.146). Likewise, there were no racial differences in plasma [nitrite], although there was a large but non‐significant effect size for the increase from HWI (*η*_p_^2^ = 0.177; Figure 4d).

### 3.6 Thermoregulatory, cardiovascular and perceptual responses

The acute thermoregulatory, cardiovascular and perceptual responses throughout H1 and H10 are presented in Table 4. During HWI, *T*_core_ increased by ∼0.9°C similarly throughout H1 and H10. The only significant main effect of HWI was for *T*_skin_, which was lower during H10 than H1 (*P* = 0.043; *η*_p_^2^ = 0.209), and there were significant main effects of race, whereby *T*_skin_ (*P* = 0.034; *η*_p_^2^ = 0.225), SBP (*P* = 0.042; *η*_p_^2^ = 0.210) and TS (*P* = 0.030; *η*_p_^2^ = 0.236) were lower during HWI in SA than WE. However, there were no race × HWI interaction effects (all *P* ≥ 0.107), and the only significant HWI × race × time interaction was for TC (*P* = 0.027; *η*_p_^2^ = 0.182), whereby TC was reduced at 60 min of H10 compared with H1 in WE (*P* = 0.026) but increased in SA (*P* = 0.009), resulting in a significant difference at this time point between WE and SA (*P* = 0.010).

**TABLE 4:** Acute thermoregulatory, cardiovascular and perceptual responses throughout the first (H1) and 10th (H10) hot water immersion sessions for groups of White European and South Asian descent.

Parameter	Racial group	H1			H10		
		0	30	60	0	30	60
*T*_core_, °C	WE	37.3 ± 0.2	38.1 ± 0.3	38.2 ± 0.3	37.4 ± 0.3	38.0 ± 0.3	38.2 ± 0.4
	SA	37.4 ± 0.2	38.0 ± 0.3	38.2 ± 0.3	37.5 ± 0.2	38.2 ± 0.3	38.4 ± 0.3
*T*_skin_, °C	WE	32.4 ± 0.5	39.0 ± 0.6	36.0 ± 0.7	32.2 ± 0.3^*^	38.8 ± 0.5^*^	35.4 ± 1.0^*^
	SA	32.1 ± 0.2^#^	38.7 ± 0.3^#^	35.6 ± 0.8^#^	32.2 ± 0.2^*^, ^#^	38.7 ± 0.2^*^, ^#^	35.2 ± 0.7^*^, ^#^
SBP, mmHg	WE	124 ± 4	122 ± 5	123 ± 6	124 ± 5	122 ± 5	122 ± 6
	SA	121 ± 6^#^	116 ± 6^#^	115 ± 5^#^	121 ± 4^#^	119 ± 7^#^	119 ± 6^#^
DBP, mmHg	WE	75 ± 6	59 ± 8	60 ± 8	73 ± 5	57 ± 6	57 ± 7
	SA	77 ± 5	61 ± 5	62 ± 3	76 ± 5	61 ± 5	63 ± 4
MAP, mmHg	WE	91 ± 4	80 ± 6	81 ± 6	90 ± 4	79 ± 5	79 ± 6
	SA	92 ± 4	80 ± 5	79 ± 3	91 ± 4	80 ± 5	81 ± 3
HR, beats/min	WE	71 ± 10	106 ± 6	95 ± 8	71 ± 10	102 ± 10	89 ± 5
	SA	73 ± 9	103 ± 10	92 ± 6	74 ± 6	105 ± 10	91 ± 8
TC, 0 to 30	WE	25.8 ± 3.6	16.2 ± 6.6	22.7 ± 2.9	25.0 ± 3.8	19.1 ± 5.1	25.4 ± 3.3^‡^, ^^^
	SA	24.8 ± 4.2	17.5 ± 6.1	24.1 ± 3.4	24.7 ± 3.2	18.1 ± 4.5	20.8 ± 3.5^‡^, ^^^
TS, 0 to 30	WE	17.0 ± 3.4	24.6 ± 3.8	18.8 ± 1.5	16.3 ± 2.5	23.9 ± 2.5	18.7 ± 2.2
	SA	16.8 ± 1.9^#^	21.4 ± 4.0^#^	17.1 ± 2.5^#^	14.9 ± 2.0^#^	20.5 ± 3.0^#^	17.1 ± 2.6^#^

*Notes*: Data are the mean ± SD. Abbreviations: DBP, diastolic blood pressure; HR, heart rate; MAP, mean arterial pressure; SA, South Asian; SBP, systolic blood pressure; TC, thermal comfort; *T*_core_, rectal temperature; TS, thermal sensation; *T*_skin_, mean skin temperature; WE, White European.

* Significant main effect of hot water immersion.

^#^ Significant main effect of race.

^‡^ Significant difference between H1 and H10 within racial group.

^^^ Significant difference between WE and SA at specific time point.

## 4 DISCUSSION

This is the first study to compare the effects of repeated HWI on microvascular function and glycaemic control between individuals of WE and SA descent. The main findings were that repeated HWI increased microvascular responses to LH to a similar extent in WE and SA. In contrast, although early‐phase glucose concentrations during the OGTT were reduced solely in SA, insulin sensitivity was unchanged in either racial group. This is in partial agreement with our hypothesis that HWI would elicit beneficial effects on microvascular function and glycaemic control in both WE and SA, but that these improvements would be augmented in SA. Together, these results suggest that HWI might have the potential to improve certain outcome measures related to microvascular function and glycaemic control, particularly in SA males, who exhibit increased risk profiles, although several measures were also unchanged, as described below.

In comparison to WE, impaired vascular responses to a variety of stimuli have been observed in the SA population (Chambers et al., 1999; Murphy et al., 2007; Roberts et al., 2023; Shantsila et al., 2011). This suggests that SA exhibit vascular dysfunction, which has been proposed to contribute to heightened CVD risk and mortality (Lind et al., 2011; Yeboah et al., 2009). In the present study, cutaneous forearm and great toe PORH and LH responses were principally similar between WE and SA, with the only differences being a lower SA baseline forearm CVC, which resulted in a greater forearm PORH index. However, similar CVC responses between WE and SA using the same microvascular assessments as in this study have been demonstrated previously (Bellini et al., 2025). Despite PORH being unaffected by HWI, both forearm and great toe CVC during 42°C LH, in addition to great toe CVC during 44°C LH, were greater Post HWI compared with Pre, increasing by a similar extent in both racial groups. Great toe CVC increasing at both LH temperatures, in contrast to forearm CVC increasing only at 42°C, might be related to the HWI protocol. Specifically, the lower limbs were immersed for the entire 60 min HWI exposures, whereas the upper limbs were immersed only for the initial 30 min, resulting in the forearm site receiving half of the total immersion duration compared with the great toe. Importantly, Romero et al. (2022) postulate that responses exhibited during and following acute heat exposure underpin chronic adaptations elicited by HT. Therefore, given that the HWI protocol acutely increases great toe but not forearm CVC responses to LH (Bellini et al., 2025), this might also explain the site‐specific differences between measurement locations. Moreover, despite potential population‐wide differences in CVD risk and vascular function between WE and SA, the comparable improvement might be explained by the similar acute responses to HWI between racial groups (Bellini et al., 2025). Lastly, although HT has the potential to reduce BP (Pizzey et al., 2021), resting BP was unchanged following HWI. This contrasts with similar‐duration (Blankenship et al., 2025; Hoekstra et al., 2018) and longer‐term HT interventions (Brunt, Howard, Francisco, et al., 2016; Ely, Francisco, Halliwill, et al., 2019; Ruiz‐pick et al., 2025), although it is in accord with some experimental studies (James et al., 2024; Kaiser et al., 2025). These differing findings might be explained, in part, by the magnitude by which *T*_core_ increased during HT, in that the 0.9°C increase observed in the present study is less pronounced than other HT interventions (Brunt, Howard, Francisco, et al., 2016; Ely, Francisco, Halliwill, et al., 2019; Hoekstra et al., 2018; James et al., 2024; Kaiser et al., 2025).

The LH‐specific improvement by HWI might be attributed to the underlying mechanistic differences from PORH. Following an initial increase, skin blood flow plateaus during prolonged LH and is primarily NO dependent (Kellog et al., 1998; Minson et al., 2001), whereas sensory nerves and endothelium‐derived hyperpolarizing factors have been implicated in the PORH response (Larkin & Williams, 1993; Lorenzo & Minson, 2007). There is evidence that chronic HWI can improve microvascular function through NO‐dependent dilatation (Brunt, Eymann, Francisco, et al., 2016), whereby increases in NO bioavailability have been suggested to contribute to beneficial vascular adaptations (Brunt & Minson, 2021). Indeed, transient increases in plasma [nitrite], a circulating marker of endothelial nitric oxide synthase‐derived NO (Lauer et al., 2001), have been observed following acute HT (Hoekstra et al., 2018; Su et al., 2025). In the present study, resting plasma [nitrite] was unaffected by chronic HWI, which is supported by previous research involving an HWI intervention of similar duration (Hoekstra et al., 2018). Given that the specific HWI protocol we used does not acutely increase plasma [nitrite] (Bellini et al., 2025) and that increases appear to be related to the strain experienced during HT (Hoekstra et al., 2021; Su et al., 2025), this suggests that greater durations or temperatures of HWI might be required to elicit chronic elevations. The increase in CVC during LH from HWI might also be explained by differences between systemic circulating venous plasma levels of nitrite and local cutaneous NO bioavailability, in addition to alternative mechanisms, such as enhanced endothelial sensitivity to NO through reduced oxidative stress (Brunt & Minson, 2021). However, given that techniques such as microdialysis or iontophoresis were not used, the mechanistic pathways underlying improvements in microvascular function could not be determined. Nonetheless, the time frame in which HWI elicited increases in microvascular function is of interest, because studies demonstrating beneficial improvements following HT (Brunt, Eymann, Francisco, et al., 2016; Carter et al., 2014b; Green et al., 2010) have used longer‐herm HT protocols (≥24 sessions), whereas shorter‐term HT interventions typically do not affect the microvasculature (Francisco et al., 2017; James et al., 2024). However, heterogeneity in the heating duration, temperature, area of heating and time between the final heating session and measurement of outcome variables result in challenges when comparing the magnitude of effect across interventions.

Although there were similar effects on microvascular function between racial groups, glucose iAUC 0–30 min, alongside [glucose] (at 15 and 30 min) and peak [insulin], were reduced following HWI in SA but not WE. Numerous studies have also identified metabolic benefits from chronic HT, such as improved insulin sensitivity (James et al., 2023; Hesketh et al., 2019), lowered Hb_A1c_ (Hooper, 1999), reduced [glucose] or [insulin] both in a fasted state (Koçak et al., 2020; Hoekstra et al., 2018; Pallubinsky et al., 2020; James et al., 2023) and during glucose challenges (Ely, Clayton, McCurdy, et al., 2019; Fuchs et al., 2025; Hesketh et al., 2019), although glycaemic control has also remained unchanged (Blankenship et al., 2025). The observed reduction of [glucose] during the OGTT, which resulted in lowered glucose iAUC 0–30 min and peak [insulin], could indicate a favourable shift to the glucose–insulin dynamics within SA. Although these metabolic adaptations might reflect improved insulin signalling or enhanced glucose clearance or uptake (An et al., 2023; Goff, 2019), it should be noted that overall insulin sensitivity was unchanged in both racial groups. Furthermore, given that a hyperinsulinaemic–euglycaemic clamp was not used, which is the gold‐standard method to quantify whole‐body insulin sensitivity, further research is needed to understand the factors mediating beneficial metabolic adaptations from HT. Nonetheless, proposed underlying physiological mechanisms include the upregulation of heat shock proteins (Faulkner et al., 2017), improvements to vascular endothelial function or skeletal muscle blood flow (Baron et al., 1994; Brunt & Minson, 2021) and reduced inflammatory status (Hoekstra et al., 2020). Although inflammation has been highlighted as contributing to both vascular and metabolic dysfunction (Butkowski & Jelinek, 2017; Libby et al., 2002), both microvascular function and glycaemic control were improved independent of changes in inflammatory profile. Similar HWI interventions have also had no effect on markers of inflammation (Hoekstra et al., 2018; James et al., 2023), although there was a non‐significant but ‘large’ effect size observed for a reduction in the IL‐6:IL‐10 ratio. Lastly, beneficial metabolic adaptations have also been conferred from 10 HWI sessions over 14 days (Hoekstra et al., 2018; James et al., 2024), a shorter time frame than previously outlined for improvements in microvascular function. The minimal weekly dose of HT required to elicit improvements to microvascular function and glycaemic control warrants investigation, with implications for reducing cardiometabolic risk profiles.

There is evidence that individuals with impaired metabolic health might experience a heightened benefit from HT. For instance, correlation analyses have indicated that participants with higher baseline insulin concentrations experience a greater reduction from HWI (Hoekstra et al., 2018; James et al., 2023). Within the present study, SA had elevated [glucose] and [insulin] during the OGTT, which aligns with population‐wide data suggesting that impaired glycaemic control contributes to elevated risk of T2DM in the SA population (Meeks et al., 2016). This metabolic dysfunction has been attributed, in part, to lower aerobic fitness levels, elevated body fat and greater inflammatory levels (Celis‐Morales et al., 2013; Chandalia et al., 2003; Goff, 2019). In the present study, the only differences in these outcomes between racial groups were an elevated IL‐6:IL‐10 ratio in SA, indicating a more pro‐inflammatory status (Viana et al., 2014). For the two metabolic outcomes where SA‐specific improvements were identified (i.e., glucose iAUC 0–30 min and peak insulin), when ‘Pre’ values were included as a covariate, our results indicate that distinct responses to HWI between racial groups were not driven by baseline metabolic status. Therefore, this appears to be mediated by distinct physiological mechanisms, whereby enhanced vascular endothelial function, upregulation of heat shock proteins and increased insulin‐independent glucose uptake have been proposed as contributing to improved glucose disposal or insulin signalling (An et al., 2023; Brunt & Minson, 2021; Faulkner et al., 2017). Nonetheless, although overall insulin sensitivity was unchanged from HWI in both racial groups, it appears that populations with more unfavourable health profiles might receive the greatest benefit from HT. Indeed, metabolic benefits from HT have occurred primarily within clinical populations (Ely, Clayton, McCurdy, et al., 2019; Hooper, 1999; James et al., 2023), overweight or obese individuals (Hoekstra et al., 2018; Koçak et al., 2020; Pallubinsky et al., 2020) and older adults (Fuchs et al., 2025). In contrast, the glucose and insulin data observed in WE participants within the present study do not reflect metabolic dysfunction through hyperglycaemia or insulin resistance, in part because they were young, healthy, recreationally active males. Therefore, given the comparably favourable health status of the WE racial group, longer HT interventions might be required to elicit beneficial metabolic adaptations within apparently healthy WE male populations. Lastly, given that HWI can acutely affect glycaemic control (Leicht et al., 2019; Maley et al., 2019, 2023), it is possible that this might explain, in part, the differences in chronic adaptations observed in this study.

The main limitation of this research pertains to the lack of a thermoneutral condition or a time‐matched control group, because a pre–post experimental design was used. Given that the main experimental question focused on comparing the effects of HT between racial groups, the WE group served as a ‘reference’ group relative to SA, because HT research has historically included predominantly WE individuals. Nonetheless, a control group would have increased the robustness of these results, although HWI studies of longer duration, which have included a thermoneutral control, have reported no change in a multitude of clinical measures (Brunt, Howard, Francisco, et al., 2016). Another limitation surrounds the number of participants included within each racial group. Given the dual outcome measures within the present study, the a priori sample size calculation was conducted using the smaller effect observed within previous HT research on microvascular function (Brunt, Eymann, Francisco, et al., 2016) compared with glycaemic control (Hoekstra et al., 2018). However, this effect size was derived from an 8 week, more thermally intensive HWI protocol using the highly controlled microdialysis technique, and using this effect size is likely to have overestimated the magnitude of effect achievable within the present study when considering the shorter and less thermally intensive intervention. Therefore, the present study might be underpowered to detect smaller changes in microvascular function. Moreover, given that this study is, to our knowledge, the first to compare the effect of HWI between distinct racial groups, we aimed to include a breadth of outcome measures. Although race‐specific effects were observed within a few select outcomes related to glycaemic control, it should be noted that the majority of microvascular and glycaemic control outcome measures were either unaffected by HWI or the effects did not differ between racial groups. Consequently, these race‐specific effects should be interpreted as hypothesis generating, requiring future confirmation in adequately powered studies.

Our results should be considered only in the context of young and healthy WE and SA males, given that females and clinical groups were not included in this study. Although the inclusion of females would have increased the generalizability of this study, females were excluded with the aim of reducing potential biological variability, which could confound the new comparisons between racial groups. Although menstrual effects might have only a small effect on vascular function (Williams et al., 2020), there is some evidence that the broader hormonal environment, encompassing both endogenous fluctuations and exogenous exposures, might influence microvascular responses (Charkoudian et al., 1999; Su et al., 2025; Turner et al., 2023) and result in potential sex differences (Turner et al., 2023). Importantly, research characterizing hormonal influences and sex differences has primarily involved females of WE descent, with limited investigation of whether potential hormonal influences or sex differences occur across racial groups. However, there is some evidence that mechanisms mediating vascular dysfunction within a non‐White racial group, specifically individuals of Black African descent, might be sex dependent (Brothers et al., 2019; Patik et al., 2018), suggesting possible interactions between ‘race’ and ‘sex’ on vascular physiology not yet characterized within SAs. Therefore, although we view the specific inclusion of SA, a historically under‐researched population, as a novel strength of this study, future research should aim to investigate the interaction between race and sex as biological variables across the health and age spectrum. Additionally, although skin measuring and marking were used to replicate the sampling location, biological variability can result in moderate‐to‐poor repeatability measures of CVC when using single‐fibre measurement probes (Roustit et al., 2010). Despite this variability, both forearm and great toe CVC plateaus during LH increased from HWI, with prior research also demonstrating improvements to microvascular function from HWI as previously highlighted. Lastly, the relatively short duration of the intervention should be considered. Although 10 HWI sessions have commonly been used (Hoekstra et al., 2018; James et al., 2024, 2023), longer‐term HT interventions have reported significant improvements occurring after 10 HWI sessions (Brunt, Howard, Francisco, et al., 2016; Ely, Clayton, McCurdy, et al., 2019), supporting the epidemiological data suggesting that more frequent HT results in greater health benefits (Laukkanen et al., 2015).

## 5 CONCLUSION

In conclusion, 10 HWI sessions improved microvascular function during cutaneous LH independent of racial background in WE and SA males. In contrast, despite elevated glucose and insulin during the OGTT, early‐phase glucose concentrations were reduced in SA following the HWI intervention, although established markers of overall glycaemic control (i.e., insulin sensitivity) were unchanged in both racial groups. Together, these results provide support that HWI might elicit beneficial health adaptations, which is especially relevant for SA males, who more typically present with elevated endothelial and metabolic dysfunction.

## AUTHOR CONTRIBUTIONS

David Bellini, Alex Lloyd, Christof A. Leicht, Stephen J. Bailey, Lewis J. James and Matthew J. Maley were involved in the study conception and design. David Bellini conducted the experiments. All authors assisted in the interpretation of the data, contributed to drafting and revising the written work, approved the final version of the manuscript and agree to be accountable for all aspects of the work in ensuring that questions related to the accuracy or integrity of any part of the work are appropriately investigated and resolved. All persons designated as authors quality for authorship, and all those who qualify for authorship are listed.

## ACKLOWLEDGEMENTS

This research was supported by the National Institute for Health and Care Research (NIHR) Leicester Biomedical Research Centre. The views expressed are those of the authors and not necessarily those of the NHS, the NIHR or the Department of Health. We thank the Undergraduate and Masters students who assisted with data collection and the participants for giving their commitment and time to this research project.

## CONFLICT OF INTEREST

The authors declare no conflicts of interest.

## Data Availability

Data are available upon reasonable request from the corresponding author.
